# Case report: a brain abscess in a disaster zone- beyond the call of duty

**DOI:** 10.1186/s40696-015-0003-2

**Published:** 2015-06-08

**Authors:** Giora Weiser, Joseph Mendlovic, David Dagan, Dov Albukrek, Manor Shpriz, Ofer Merin

**Affiliations:** 1 Medical Corps, Israel Defense Forces, Tel Hashomer, Israel; 2 Shaare Zedek Medical Center, 12 Shmuel Bait street, Jerusalem, 93722 Israel; 3 Israel Ministry of Health, Jerusalem, Israel; 4 Reuth Rehabilitation Center, Tel Aviv, Israel

**Keywords:** Field hospital, Brain abscess, Disaster

## Abstract

We present a case of a child with a suspected brain abscess treated by a military field hospital in post-typhoon Philippines. We review our intervention and decision process both at the field hospital and following his transfer to a referral center. These interventions were critical for his successful outcome.

## Background

Brain abscesses present as a life threatening disease requiring prompt diagnosis and emergent treatment with wide range antibiotics and possible neurosurgical intervention. The most common cause of brain abscesses requiring neurosurgical drainage is Staphylococcus aureus (SA) [1]. Without such aggressive interventions and treatment, morbidity and mortality are the rule.

We present a case of a brain abscess in a 4 year old male, diagnosed at the Israel Defense Forces Field Hospital (IDFFH) in the Philippines following a typhoon and his management within and following transfer. Such cases require advanced medical abilities not usually available in primary settings or areas affected by natural disasters. Although an advanced field hospital was available, its resources were generally aimed at offering treatment for masses and less on a concentrated effort on a single complicated patient.

## Case presentation

On November 8th 2013, Typhon “Haiyan” passed through central Philippines. In its aftermath, more than 5000 people were killed. Severe damage was caused to the Philippine infrastructure, especially in rural areas. The IDFFH, by invitation of the Philippine government, set up its facility adjacent to the “Severo Verallo” district primary hospital in Bogo city on the island Cebu. The medical team was confronted mainly with primary care related to the destruction and loss of medical abilities in rural areas.

A four year old male was transferred by his family from another small medical facility, where he was treated for a febrile illness and subsequently developed intractable seizures. Treatment was started with Ceftriaxone. His past medical history was unremarkable. His parents noted a head injury a month earlier.

On arrival he was seizing with general tonic-clonic seizures that were stopped with intravenous (IV) midazolam. Following the cessation of the seizures his examination was remarkable for positive meningeal signs and a clear left abducens nerve palsy as well as hyperreflexia in his feet. He was hemodynamically stable and no other remarkable findings were noted. There were no heart murmurs on exam.

Trying to seek a more accurate diagnosis and following a fundoscopic exam that was normal, a lumbar puncture was performed. It showed a pleocytosis with 1200 white blood cells/dl. The accuracy of the mobile laboratory at examining Cerebral Spinal Fluid (CSF) was questionable. However, cultures were available at the time. CSF chemistries were unavailable.

The local medical facility had limited resources, which do not include an intensive care unit. In light of his grave situation and with no immediate transfer option, we assembled equipment in one of the hospital rooms to give more intensive care.

Treatment was continued with Ceftriaxone. Acyclovir was added to cover herpes infections. Due to his abducens palsy and clinically suspected elevated intracranial pressure (ICP), mannitol was administered.

His overnight observation was uneventful with no further seizures.

The following morning he was transferred to the central hospital in the island’s main city Cebu.

CSF culture showed a growth of SA. There was no option to define whether this strain was methicillin sensitive (MSSA) or resistant (MRSA). However, several other cultures taken from the local population surprised as being MRSA.

A follow up visit the following day found the child lethargic yet stable, with no significant improvement.

Adding vancomycin to his regimen would be an additional cost the family could not afford. Recognizing this need, we provided the hospital with vancomycin for the child.

Another follow-up with the child found him in the same state. He had undergone computerized tomography (CT) of the brain but no official results were reported. These typically were available 2–3 days following the exam.

The on call pediatrician allowed us access viewing the CT in the radiology department.

**Figure 1:**
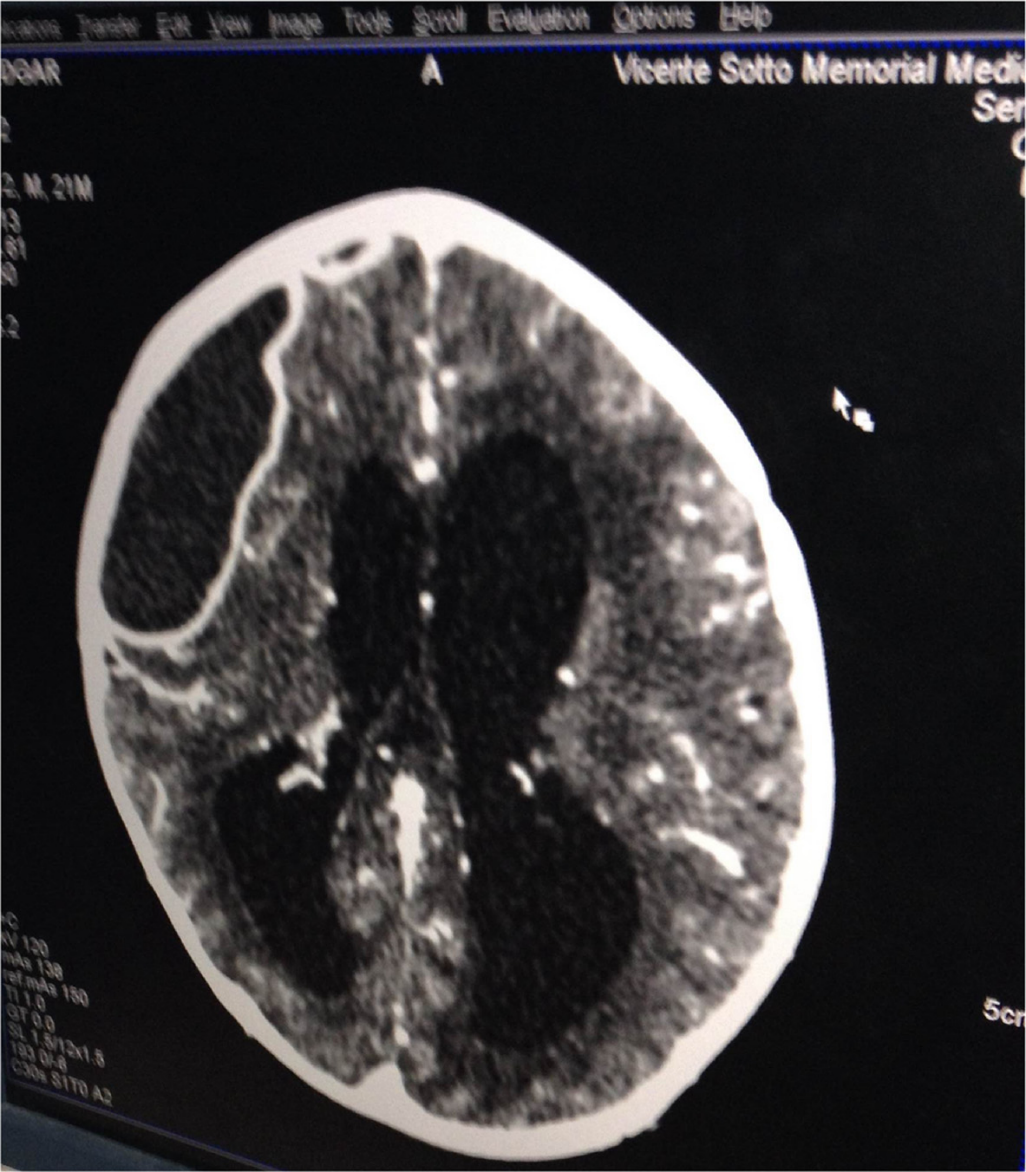
**A picture (taken with a smartphone) of the child’s Brain CT showing an obvious cystic mass in the right fronto-temporal region and seemingly enlarged ventricles.**

The CT showed an obvious right frontal cystic lesion, suspected as an abscess along with enlarged ventricles (Figure 1). Although no radiologist was available, we urged the local pediatric staff to alert the neurosurgeons as to this child’s suspected abscess.

Following our visit, the child was operated on within several hours. An abscess was found and evacuated. He showed a quick and marked improvement.

Follow up emails show him to now be fully alert and without any neurological deficits. He received a month of IV antibiotic treatment and a ventriculoperitoneal shunt was inserted successfully.

## Discussion

Under the unique conditions in a disaster zone, our decision to treat a child with a complex condition altered the expected course of his illness. It deviates from classic disaster medicine approach of giving the maximal patients the minimum needed. However, when the team and available equipment allow, such children should be given a chance for recovery and survival. Such decisions are not made lightly and involve both medical and administrative aspects.

On arrival at a disaster site, early contact should be created with local health representatives to assess both the ideal utility of the relief teams abilities as well as an understanding of local and referral center abilities. The IDFFH achieved this by sending an early team to assess the situation and enable maximal impact of the relief team.

Relief teams in general and the IDFFH specifically are based on personnel who are gathered following an unexpected event. Most of the teams professional activity is at medical facilities. It may be advised to create an available consulting group that will be available at the home country of the relief team to offer telecommunication help for both the relief team and health facilities at the disaster site.

In this case, several decisions may be questioned:

As CT scan was not available, should one preform an LP examination on a child with neurological deficits which might cause deleterious outcomes? A normal fundoscopy does not rule out an impending herniation [2].

Should steroids or Mannitol be given? Mannitol is not well studied especially in cases of brain abscesses [3]. Steroids have limited evidence of usefulness and assuming his quick transfer to a referral center pending a clear diagnosis, this treatment was withheld [4].

When should this child be transferred? Should we try to stabilize his condition in a suboptimal environment or to refer early in the course? The referral center was approximately a 5 hour drive, on bad roads, and with minimal communication. Therefore the decision was made to postpone referral till the next morning, and to establish a temporary Intensive care area.

All these decisions were made jointly by the pediatric team trying to achieve a diagnosis and in order to improve the treatment the child would receive.

In disaster areas medical teams are taught to shift their concentration from the single patient to population based. Referring to resource use in disaster areas the World Health Organization (WHO) states: “It is ethical for a physician not to persist, at all costs, in treating individuals “beyond emergency care”, thereby wasting scarce resources needed else-where. The decision not to treat an injured person on account of priorities dictated by the disaster situation cannot be considered a failure to come to the assistance of a person in mortal danger. It is justified when it is intended to save the maximum number of individuals” [5].

Concentrating on a single complicated patient who might use valuable resources could be at the expense of many others in need. The attention and resources needed to treat this specific child were well beyond the usual given in such a disaster area. Starting from the personel needed to improvise an “intensive care”, through the transportation team, and the “follow up” team that insisted on getting the exact diagnosis and accordingly the proper treatment. These combined efforts saved his life, against all odds in such an environment. The joint decision made by the pediatric team, together with the administrative personnel was done with the understanding that the burden of treating this child would be on the shoulders of the medical staff and not at the expense of other children.

## Conclusion

In conclusion, medical teams confronted with disaster areas, should find the right balance between population based treatment and the single patient. Continuing the intervention beyond the immediate stabilization and evacuation, but also to the follow up given in the referral center in this specific patient changed the natural expected course. Such extra care and involvement can be beyond the usual call of duty but may significantly alter the child’s outcome.

## Consent statement

As this is a descriptive report without any identifiable details and due to complete lack of communication with the patient and his family, No consent is available. Repeated attempts to reach the family or their representatives were unsuccessful.

## References

[1] Sheehan JP, Jane JA, Ray DK, Goodkin HP (2008). Brain abscess in children. Neurosurg Focus.

[2] van Crevel H, Hijdra A, de Gans J (2002). Lumbar puncture and the risk of herniation: when should we first perform CT?. J Neurol.

[3] Grände PO, Romner B (2012). Osmotherapy in brain edema: a questionable therapy. J Neurosurg Anesthesiol.

[4] Ackerman AD, Singhi S (2010). Pediatric infectious diseases: 2009 update for the Rogers’ Textbook of Pediatric Intensive Care. Pediatr Crit Care Med.

[5] WMA Statement on Medical Ethics in the Event of Disasters. Downloaded from: http://www.wma.net/en/30publications/10policies/d7/

